# Gold Nanoparticles Modified With Polyethyleneimine Disturbed the Activity of Drug-Metabolic Enzymes and Induced Inflammation-Mediated Liver Injury in Mice

**DOI:** 10.3389/fphar.2021.706791

**Published:** 2021-07-15

**Authors:** Hanqing Chen, Shuang Zhou, Meilin Zhu, Bing Wang, Wei Chen, Lingna Zheng, Meng Wang, Weiyue Feng

**Affiliations:** ^1^ Department of Gastroenterology, Guangzhou Digestive Disease Center, Guangzhou First People’s Hospital, School of Medicine, South China University of Technology, Guangzhou, China; ^2^ CAS Key Laboratory for Biomedical Effects of Nanomaterials and Nanosafety, Institute of High Energy Physics, Chinese Academy of Sciences (CAS), Beijing, China; ^3^ University of Chinese Academy of Sciences, Beijing, China; ^4^ Institute of Physical Science and Information Technology, Anhui University, Hefei, China

**Keywords:** gold nanoparticles, drug-metabolic enzymes, hepatic transporters, cytochrome P450, liver inflammation

## Abstract

Gold nanoparticles (GNPs) have been used as a potential bioactive platform for drug delivery due to their unique optical and thermal characteristics. Liver is the main organ in orchestrating physiological homeostasis through metabolization of drugs and detoxification of exogenous substances. Therefore, it is crucial to deeply understand the mechanism of nanoparticle–liver interaction and the potential hepatic effects of GNPs *in vivo*. In this study, we studied the hepatic impacts of the intravenously injected polyethyleneimine (PEI)-modified GNPs (PEI-GNPs) on the expression of hepatic drug-metabolic enzymes and sterol responsive element binding protein 1c (SREBP-1c)-mediated *de novo* lipogenesis in mice for 24 h and 1 week. PEI-GNP accumulation in the liver is associated with increased liver inflammation, as evidenced by the gene expression of pro-inflammatory cytokines. Moreover, the GNP-induced hepatotoxicity in mice is partly due to liver inflammation–triggered disruption in the function of drug-metabolic enzymes, including hepatic uptake and efflux transporters, cytochrome P450 (CYP450), and UDP-glucuronosyltransferases (UGTs). The study provides evidence that it is necessary to consider the nanomaterial–liver interaction and manipulate the surface chemistry of GNPs prior to biomedical application of nanoparticles.

## Introduction

With the development of nanotechnologies over the recent decades, a variety of nanomaterials including gold nanoparticles (GNPs) have been explored in medical fields, including drug delivery, diagnosis, imaging, and even cancer therapy, due to their special physicochemical properties and highly tunable natures (Bromma and Chithrani, 2020; Nutan et al., 2020; Song et al., 2010; Yang et al., 2021). The U.S. Food and Drug Administration (FDA) requires nanoparticles that are used as the pharmacological drugs for the potential medical benefits, and that should be metabolized or excreted from the body (Poon et al., 2019). With the widespread biomedical utility for the drug delivery system, it is of great interest to understand the pharmacokinetics and toxicological effect of GNPs *in vivo* (Zhou S. et al., 2020).

In order to increase the loading capacity and improve the stability of the delivery platform, amino silanes or cationic polymers, such as polyethyleneimine (PEI), are widely used to functionally modify nanomaterials, which can electrostatically interact and effectively load with negatively charged biomolecules, such as drugs, nucleic acids, and proteins, to form polyelectrolyte complexes (Wang et al., 2016; Zhu et al., 2018). Recent studies have demonstrated that PEI with low molecular weight, such as 0.6, 1.2, and 1.8 kDa, showed no cytotoxicity in PANC-1, BxPC3, and HEPA-1 cells at the doses of 6–100 μg/ml for 16 h (Xia et al., 2009; Chou et al., 2018). Previous studies have demonstrated that PEIs and their derivatives have been explored as a potential multifunctional platform for drug or gene delivery (Chen et al., 2020; Goswami et al., 2020). However, lots of evidence have reported that PEIs with the sizes more than 10 kDa exhibited significant cytotoxicity through their proton sponge effect, which leads to increased proton pump activity inside the cell, enhanced osmotic swelling of endocytic compartment, and endosomal rupture–mediated cell death (Benjaminsen et al., 2013; Vermeulen et al., 2018; Li et al., 2021), indicating that modification of PEI is of utmost importance for the biomedical and clinical application of nanoparticles.

The liver is the main organ in orchestrating physiological homeostasis through metabolization of drugs and detoxification of exogenous substances (Almazroo et al., 2017). As demonstrated by the recent studies, the liver is the largest macrophage-rich organ and acts as the most prominent organ for sequestering more than 30% of the injected nanoparticles, which prevents the administrated nanoparticle delivery to the diseased tissue (Tsoi et al., 2016; Zhang et al., 2016; MacParland et al., 2017; Poon et al., 2019). In light of the current literature, due to reduced velocity through interaction with Kupffer cells and hepatic sinusoid endothelial cells, most of the targeted nanoparticles will be trapped and accumulated in the liver (Tsoi et al., 2016; Poon et al., 2019). A recent study has reported that about 72% of the administrated GNPs were found in the liver after 1 min of injection (Haute and Berlin 2017), and the content of GNPs in the liver reached the highest level after *i.v.* injection for 1 week (Li et al., 2020). After being deposited in the liver, nanomaterials especially at the sizes of 10 nm could cause alterations in liver morphometry, serum biochemistry, and the expression of drug-metabolizing enzyme genes, including Phase I and Phase II drug-metabolizing enzymes and the drug transporters (Almansour et al., 2018). Changes in the drug-metabolic enzymes are also found in the patients with nonalcoholic fatty liver disease (NAFLD) (Cho et al., 2019; Zhou S. et al., 2020), which is characterized by >5% of fat accumulation in the liver and can develop into the nonalcoholic steatohepatitis (NASH), hepatic fibrosis, cirrhosis, and hepatocellular carcinoma (HCC) (Chen et al., 2018; Chen 2020). Therefore, it is urgent to deeply understand the mechanism of nanoparticle–liver interaction and the potential hepatic effects of GNPs modified with PEI on drug-metabolic enzymes and lipid metabolism *in vivo*. In this study, we studied the hepatic impacts of the intravenously injected PEI-modified GNPs (PEI-GNPs) on the expression of hepatic drug-metabolic enzymes and sterol responsive element binding protein 1c (SREBP-1c)-mediated *de novo* lipogenesis in mice for 24 h and 1 week.

## Materials and Methods

### Materials and Reagents

Hydrogen tetrachloroaurate (III) trihydrate (HAuCl_4_, 99.99%) and silver nitrate (AgNO_3_, ≥99.8%) were obtained from Sinopharm Chemical Reagent Co. Ltd. (Beijing, China). Trisodium citrate dihydrate (Na_3_C_6_H_5_O_7_, 99%) was obtained from Alfa Aesar (Ward Hill, MA, United States). Polyethyleneimine (PEI, 10 kDa) was purchased from Aladdin Biochemical Technology Co. Ltd. (Shanghai, China). TransZol Up Plus RNA Kit was purchased from TransGen Biotech Co. Ltd. (Beijing, China). 4% paraformaldehyde was purchased from Solarbio Life Science (Beijing, China). BeyoRT™ First Strand cDNA Synthesis Kit (RNase H minus) and BeyoFast™ SYBR Green qPCR Mix were obtained from Beyotime Institute of Biotechnology (Beijing, China). Quinidine (CAS 56-54-2) was purchased from Aladdin Chemistry Co. Ltd. (Shanghai, China). The deionized water used in all the experiments was obtained from Milli-Q system (18.2 MΩ cm).

### Synthesis and Characterization of Polyethyleneimine–Gold Nanoparticles

The colloidal suspension of gold nanoparticles (GNPs) was prepared using the “citrate” method by reaction of 1% HAuCl_4_, 0.1% AgNO_3_, and 2% sodium citrate in solution under stirring, which has been reported previously (Zhou S. et al., 2020). For PEI functionalization, a quantity of 0.405 g PEI was added to the above synthesized GNP solution, and then vortexed for 30 min at room temperature. The PEI-GNPs were collected by centrifugation at 16,000 rpm for 30 min, and then resuspended in Milli-Q water. Finally, the PEI-GNP solution was cooled and stored at 4°C for further use.

Transmission electron microscope (TEM, JEOL JSM-2100, Japan) was used to characterize the morphology and size of PEI-GNPs. The hydrodynamic diameter and zeta potential were measured *via* dynamic light scattering (DLS, Zetasizer Nano ZS90, Malvern, United Kingdom). Electronic vibrations and surface functional groups of the PEI-GNPs were measured by ultraviolet-visible (UV-vis) spectroscopy (Infinite M200 Pro, Tecan, Switzerland).

### Animal Experiments

Male CD-1 (ICR) mice (7-week old, 22 ± 2 g) were obtained from Beijing Vital River Experimental Animal Technology Co. Ltd. (Beijing, China). The mice were fed with sterilized chow and deionized water *ad libitum* at a regular 12 h of dark/light cycle, and acclimatized for 1 week prior to the treatment. All the animal experiments and protocols were approved by the Institutional Animal Care and Use Committee at the Institute of High Energy Physics, Chinese Academy of Sciences (No. IHEPLLSC202008).

All the animals were randomly divided into six groups (*n* = 6 per group): (a) PBS treatment for 24 h (Control-24H group), (b) 11.5 μg-PEI-GNPs/mouse/d treatment for 24 h (11.5-PEI-GNP-24H group), (c) 23 μg-PEI-GNPs/mouse/d treatment for 24 h (23-PEI-GNP-24H group), (d) PBS treatment for 1 week (Control-1W group), (e) 11.5 μg-PEI-GNPs/mouse/d treatment for 1 week (11.5-PEI-GNP-1W group), and (f) 23 μg-PEI-GNPs/mouse/d treatment for 1 week (23-PEI-GNP-1W group). Before intravenous (*i.v.*) injection of 100 μL, the PEI-GNP suspensions were sonicated for 10 min to prevent aggregation.

### Serum Biochemical Index

For assessment of the liver function, the blood was collected and the plasma were obtained by centrifugation at 3,000 rpm for 10 min at 4°C. Alanine aminotransferase (ALT), aspartate aminotransferase (AST), and alkaline phosphatase (ALP) were measured using an automatic chemistry analyzer (Celltac, MEK-6358; Nihon Kohden Co., Tokyo, Japan).

### Histopathological Analysis

For histopathological observation, liver tissues of the mice were collected after sacrifice and then quickly fixed in 10% paraformaldehyde for 48 h at room temperature. After paraffin embedding, the liver samples were cut into 6-mm sections and mounted on the glass slides for hematoxylin and eosin (H& E) staining. The liver slides from each mouse were imaged and assessed by recording the histological lesion score, including inflammatory cell infiltration, lipid accumulation, and hepatocyte injury.

### RNA Extraction and Quantitative Real-Time PCR (qRT-PCR)

The total RNA from about 30 μg of the frozen liver samples was extracted using the TransZol Up Plus RNA kit (TransGen Biotech, Beijing, China) according to the manufacture’s protocol and our previous studies (Chen et al., 2018; Zhao et al., 2020; Zhou S. et al., 2020). The RNA was analyzed and quantified with a NanoDrop™ One/One^C^ Microvolume UV-Vis Spectrophotometer (Thermo Fisher Scientific, MA, United States). The cDNA was reverse-transcribed from 1 μg of the total RNA according to the cDNA Reverse Transcription Kit (Takara Biotechnology, Otsu, Japan), and the 20 μL reaction mixture included 10 μL of total RNA, 2 μL of 10 × RT buffer, 1 μL of 25 × dNTP mix (100 mM), 2 μL of 10 × RT random primer, 1 μL of reverse transcriptase, 1 μL of RNase inhibitor, and 3 μL of nuclease-free water. The reaction was carried out as follows: 25°C for 10°min, 37°C for 120°min, and 85°C for 5 min. cDNA samples were stored at −20°C until use. The RT-PCR was performed in the presence of BeyoFast™ SYBR Green qPCR Mix on a CFX Connect Real-Time PCR Detection System (Bio-Rad). For RT-PCR reaction conditions, the initial activation stage was performed at 95°C for 2 min, followed by 40 cycles of thermal denaturation at 95°C for 20 s, and annealing and elongation at 60°C for 30 s. Gapdh was used as the invariant control. The 2^-∆∆Ct^ method was used to calculate the relative level of mRNA in the liver of the mice with or without PEI-GNP treatment. The primers are listed in Table 1.

**TABLE 1:** List of the sequences of qPCR primers used in this study.

Genes	Forward primer sequences (5′-3′)	Reverse primer sequences (5′-3′)
*mSlc10a1*	GTT​CTC​ATT​CCT​TGC​GCC​AT	GCC​ACA​GAG​AGG​GAG​AAA​GT
*mSlc22a1*	GAA​TGT​GAC​CCT​CGC​TTG​TC	TCC​ATC​AGC​CTG​AAC​ACC​AT
*mSlc22a7*	TAG​ACC​TGT​TCC​GGA​CAT​CG	GAC​GCA​CAA​GGA​AGA​AGA​CC
*mSlco1b1*	AGG​GTA​GGC​GGT​CAT​TTG​AA	GCA​GTG​CCT​TGT​CTT​CTC​TG
*mSlco2b1*	GCC​TCA​CTA​TCC​CTC​TGG​TC	TGG​GTA​GAG​CAG​CCA​ATG​AA
*mAbcb1a*	CTT​GGT​GGC​ACA​ACA​ACT​CA	TTC​AAG​CCC​TCT​GTG​CTG​TA
*mAbcb1b*	ATG​GCT​GGA​TCA​GTG​CTC​TT	GAG​AAT​GGG​TTC​CTG​GGA​CA
*mAbcb4*	CAG​TAT​CGA​CGG​ACA​GGA​CA	CTT​CTC​GGC​CAT​AGC​GAA​TG
*mAbcc1*	CTG​CCC​TCT​TTG​CAG​TCA​TC	TCC​ATC​TCC​GAG​GAC​ATT​CG
*mAbcc2*	AGA​GCT​AGC​CTG​CTT​GGA​AA	CCT​TTG​GCT​CTG​TCC​ACC​TA
*mAbcc3*	TGA​GGA​TGC​GGT​CCT​ACT​GA	AGC​GTA​CAG​CTT​GAG​CAC​TT
*mCyp1a2*	GGA​GGC​TAA​CCA​TCT​CGT​CA	ATG​GCA​CCA​ATG​ACG​TTA​GC
*mCyp2a4*	GGC​TAC​CTT​CGA​CTG​GCT​TT	GTC​CTT​GGT​GAC​CCT​TCG​AG
*mCyp2c37*	GGC​TAC​CTT​CGA​CTG​GCT​TT	GTC​CTT​GGT​GAC​CCT​TCG​AG
*mCyp2c40*	GCC​ACA​AGG​GTC​TTC​ACA​GT	TCA​CCA​ATG​TTG​CCA​GGT​GT
*mCyp2c44*	ACC​CTC​GGG​ATT​ACA​TCG​AC	GGA​TGC​TTG​ACA​AGG​AGC​AG
*mCyp2c50*	AAC​ACA​AGG​CGC​TTC​TCA​CT	TTG​CAA​ACC​TGC​AAC​CAA​GG
*mCyp2d10*	GGC​TGG​AAG​CCT​ATG​GTT​GT	GCG​TTG​TTC​AGC​ATG​GTG​TT
*mCyp2d26*	GGC​TGG​AAG​CCT​ATG​GTT​GT	GCG​TTG​TTC​AGC​ATG​GTG​TT
*mCyp2d34*	ACG​TTC​CCA​ATA​CTC​CTG​CG	GTC​TGC​CAT​CTC​TGG​ACA​CC
*mCyp2d40*	GAA​GCC​CCT​CCG​CTT​CTA​TC	CTA​GCG​AAC​CAC​TGC​ACA​GA
*mCyp2e1*	AAA​CAG​GGT​AAT​GAG​GCC​CG	GGG​CAG​TTG​ATG​TCC​AGT​GA
*mCyp3a11*	AGG​GAT​GGA​CCT​GGT​TTC​AG	AGA​GGA​GCA​CCA​AGC​TGA​TT
*mUgt1a7c*	TGC​AAT​GGA​GTT​CCG​ATG​GT	CTG​GAG​AGG​CGC​ATG​ATG​TT
*mUgt1a6*	GAA​GCC​CCT​CCG​CTT​CTA​TC	CTA​GCG​AAC​CAC​TGC​ACA​GA
*mUgt1a1*	CCA​GTA​TGC​TCC​TAG​CTG​GC	TGC​CCG​AGT​CTT​TGG​ATG​AC
*mG6pase*	AAG​CCA​ACG​TAT​GGA​TTC​CG	ACA​GCA​ATG​CCT​GAC​AAG​ACT
*mPepck*	GTG​CTG​GAG​TGG​ATG​TTC​GG	CTG​GCT​GAT​TCT​CTG​TTT​CAG​G
*mPparα*	CAG​GAG​AGC​AGG​GAT​TTG​CA	CCT​ACG​CTC​AGC​CCT​CTT​CAT
*mSrebp-1c*	GAGCCATGGATTGCACAT TT	CTC​AGG​AGA​GTT​GGC​ACC​TG
*mFas*	GAG​GAC​ACT​CAA​GTG​GCT​GA	GTG​AGG​TTG​CTG​TCG​TCT​GT
*mScd1*	AGC​CTG​TTC​GTT​AGC​ACC​TT	CAC​CCA​GGG​AAA​CCA​GGA​T
*mTnf-α*	AAG​TTC​CCA​AAT​GGC​CTC​CC	CCA​CTT​GGT​GGT​TTG​TGA​GTG
*mIl-10*	GGT​TGC​CAA​GCC​TTA​TCG​GA	GGG​GCA​TCA​CTT​CTA​CCA​GG
*mIl-6*	CCC​AAT​TTC​CAA​TGC​TCT​CCT	GTC​TTG​GTC​CTT​AGC​CAC​TCC
*mIl-1*β	TGC​CAC​CTT​TTG​ACA​GTG​ATG	AGC​CCT​TCA​TCT​TTT​GGG​GT
*mTOR*	CCG​CTA​CTG​TGT​CTT​GGC​AT	CAG​CTC​GCG​GAT​CTC​AAA​GA
*mGapdh*	TTG​ATG​GCA​ACA​ATC​TCC​AC	CGT​CCC​GTA​GAC​AAA​ATG​GT

### Cell Culture and Treatment

The human hepatoma HepaRG cells were obtained from Beijing Beina Chuanglian Biotechnology Institute (Beijing, China). The cells were grown in an RPMI-1640 medium supplemented with 10% fetal bovine serum (FBS) and 1% penicillin/streptomycin, and maintained in humidified atmosphere of 5% CO_2_ at 37°C. After being grown in 96-well plates for 12 h at the density of 2 × 10^4^ cells/well, the cells were treated with GNPs at the concentrations of 1, 10, and 100 μg/ml in serum-free medium for 24 h with or without quinidine (QUN, 10 μM) pretreatment. The cell viability was detected by using a Cell Counting-8 Kit (CCK-8, Dojindo Laboratories).

### Statistical Analysis

All the experiments were performed three times, and the values were represented as the mean ± standard deviation (SD). The results were analyzed by GraphPad Prism software (version 8.0). The statistical significance was calculated using one-way ANOVA with Bonferroni’s multiple comparison posttest. The asterisks * and ** denote *p* < 0.05 and *p* < 0.01 compared to untreated cells, respectively.

## Results

### Preparation and Physicochemical Characterization of Polyethyleneimine–Gold Nanoparticles

**FIGURE 1:**
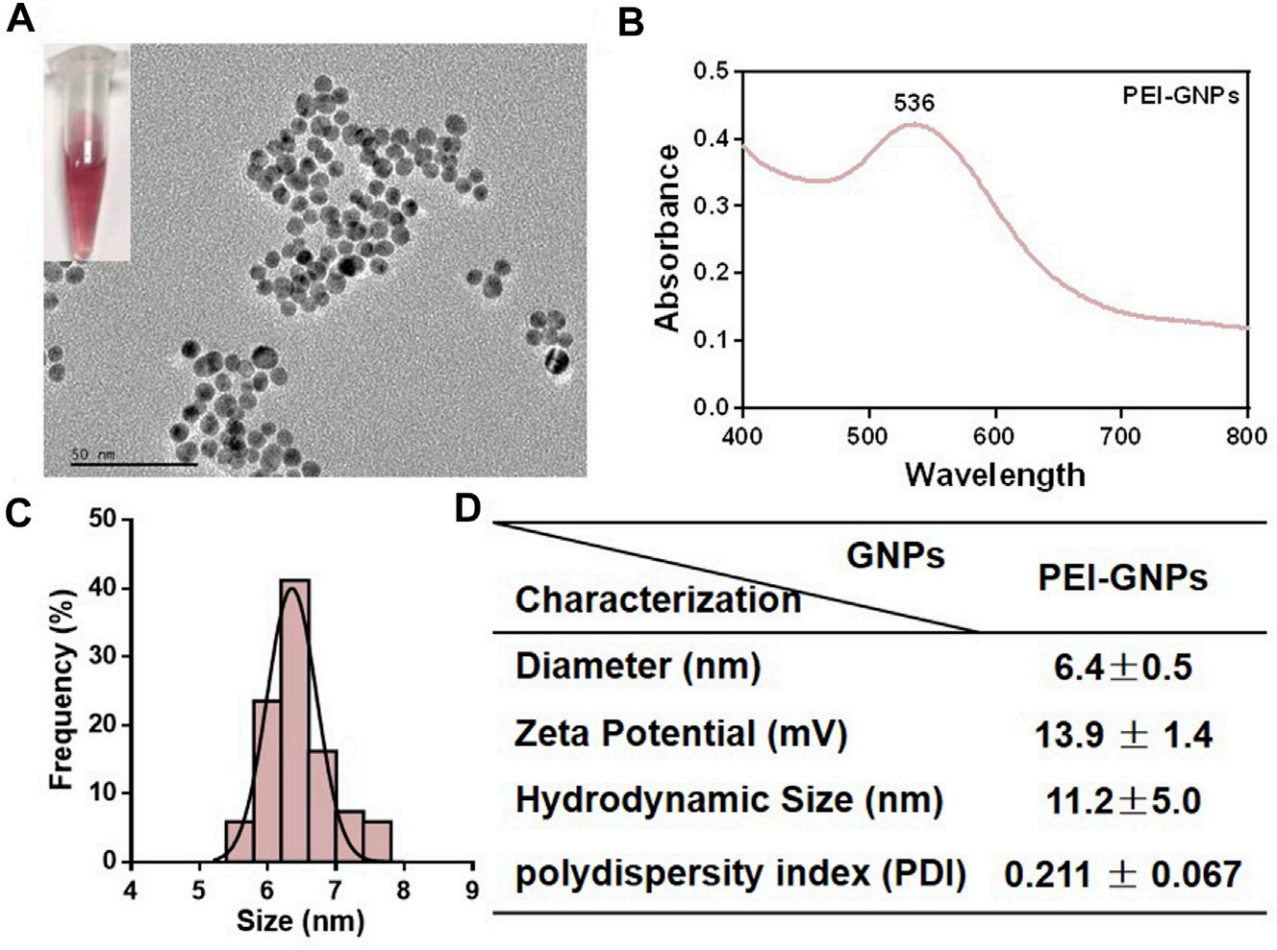
Physicochemical characterization of polyethyleneimine (PEI)-coated gold nanoparticles (PEI-GNPs). **(A)** Representative transmission electron microscopy (TEM) images of PEI-GNPs. Inserted figures: optical images of PEI-GNPs dispersed in Milli-Q water in 4°C for 1 week. **(B)** UV-Vis spectrum of PEI-GNPs. **(C)** Statistical analysis of the size distribution of PEI-GNPs in Milli-Q water measured by TEM. **(D)** The detailed information of PEI-GNPs used in this study, including diameter, zeta potential, hydrodynamic size, and polydispersity index (PDI). All the values are presented as mean ± standard deviation (SD) (*n* = 3).

The detailed information and baseline physicochemical characterization of PEI-GNPs are listed in Figure 1. A transmission electron microscope (TEM) showed that the monodispersed spherical PEI-GNPs were synthesized and well dispersed in the physiological pH solutions. The average diameter of the prepared PEI-GNPs was 6.4 ± 0.5 nm. The hydrodynamic size in Milli-Q water was 11.2 ± 5.0 nm, with a narrow size distribution [polydispersity index (PDI) = 0.211 ± 0.067], and the zeta potential was measured as 13.9 ± 1.4 mV. The UV-Vis absorption spectrum of GNPs with PEI modification (PEI-GNPs) had the characteristic absorbance peak at the wavelength of 536 nm, reflecting the surface plasmon resonance (SPR) of GNPs (Li et al., 2020; Zhou S. et al., 2020). These results indicated that PEI was successfully introduced on the surface of GNPs along with well dispersibility.

### Hepatic Effects of Polyethyleneimine–Gold Nanoparticles in Mice

**FIGURE 2:**
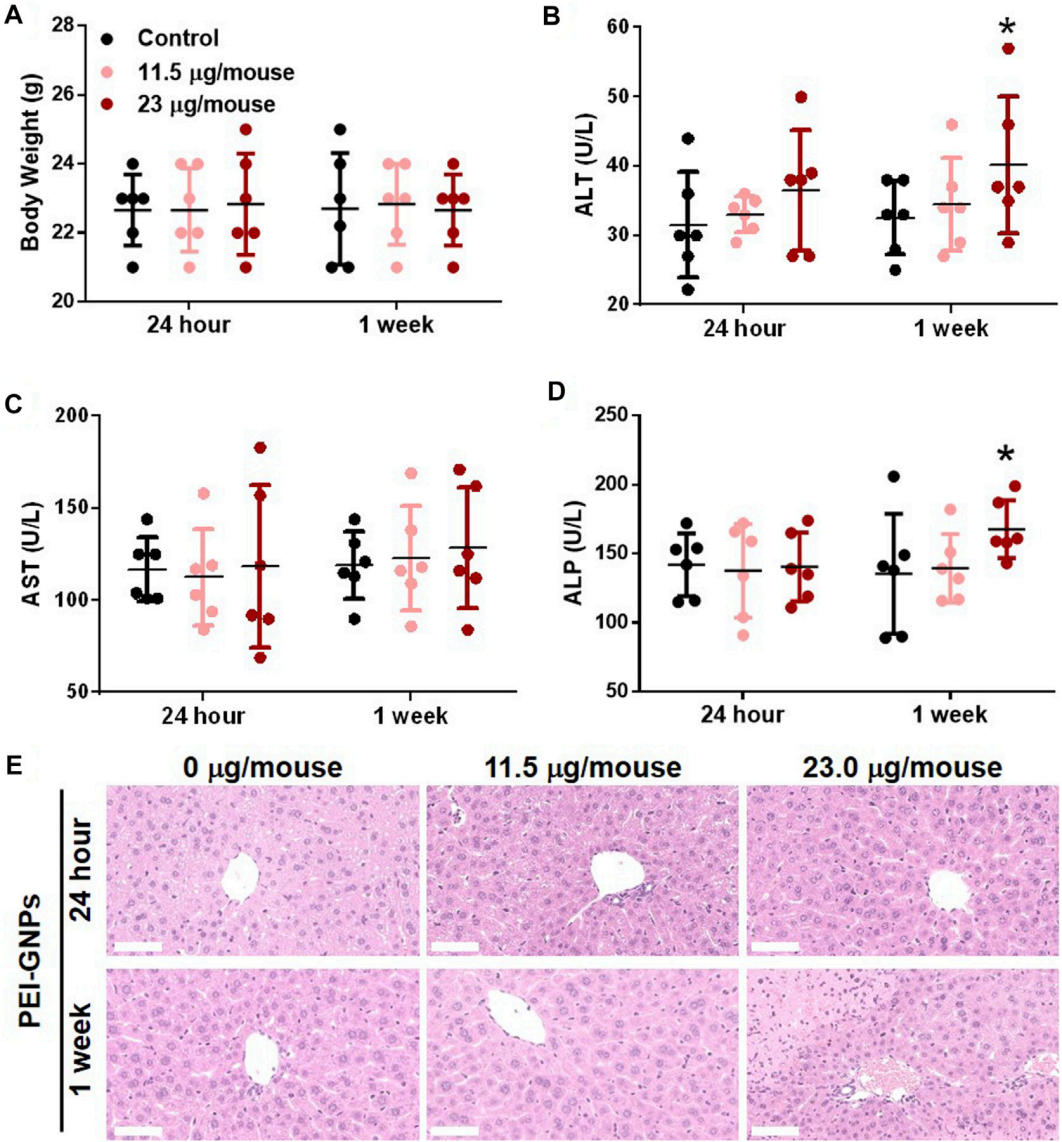
Effect of PEI-GNPs on the liver in mice after intravenous injection once for 24 h and 1 week at doses of 11.5 mg/mouse and 23.0 mg/mouse. **(A)** Average body weight of the mice treated with PEI-GNPs. Liver function tests were performed, and the plasma alanine aminotransferase [ALT, **(B)**], aspartate aminotransferase [AST, **(C)**], and alkaline phosphatase [ALP, **(D)**] levels were measured in mice treated with PEI-GNPs. All the values are presented as mean ± SD from 6 mice. *p < 0.05 vs. the mice treated with PBS. **(E)** Representative H& E staining of the liver sections to assess the histopathological injury in mice treatment with PEI-GNPs (scale bars, 50 μm).

In order to explore the potential hepatic effect of PEI-GNPs *in vivo*, PEI-GNPs were intravenously injected into ICR mice for 24 h and 1 week at the doses of 11.5 and 23 μg/mouse, respectively (Figure 2). As expected, no obvious abnormal body weight change, no significantly histological lesion, and no alteration of plasma alanine aminotransferase (ALT), aspartate aminotransferase (AST), and alkaline phosphatase (ALP) were found in mice treated with PEI-GNPs for 24 h. Furthermore, hematoxylin and eosin (H&E) staining showed that mice treated with PEI-GNPs at the dose of 23 μg/mouse for 1 week exhibited slight inflammatory cell infiltration and hepatocyte injury along with increased levels of serum ALT and ALP. However, plasma AST was comparable between all the groups. These results confirmed that PEI-GNP treatment induced liver inflammation in mice at 23 μg/mouse for 1 week.

### The Effects of Polyethyleneimine–Gold Nanoparticles on Hepatic Pro-Inflammatory Responses in Mice

**FIGURE 3:**
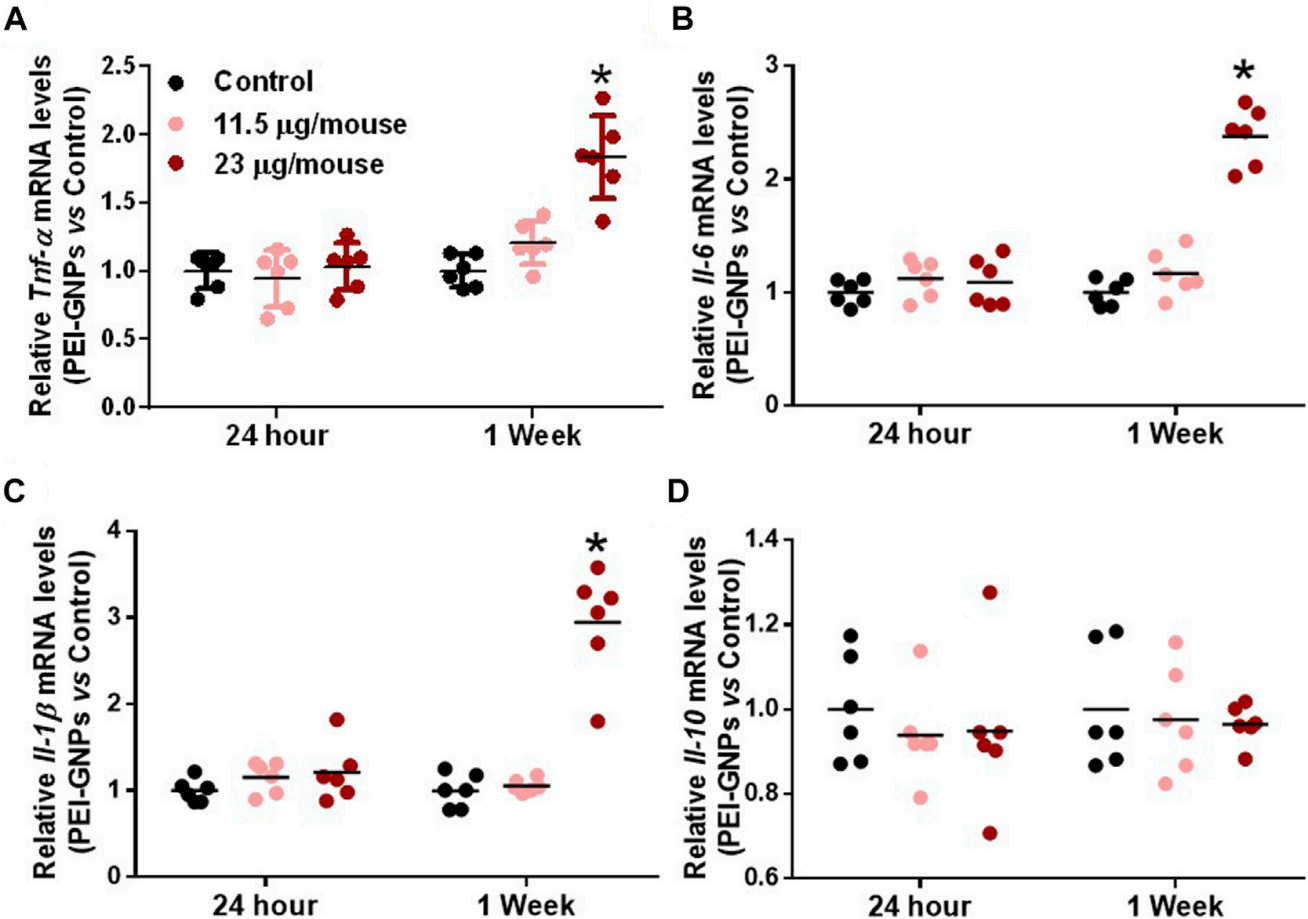
Effect of PEI-GNPs on the liver inflammation in mice. Hepatic mRNA expression of pro-inflammatory cytokines, including *Tnf-*α **(A)**, *Il-6* **(B)**, and *IL-1*β **(C)**, and anti-inflammatory cytokine, such as Il-10 **(D),** in mice treated with PEI-GNPs for 24 h and 1 week. All the values are presented as mean ± SD from 6 mice. *p < 0.05 vs. the mice treated with PBS.

In order to elucidate the underlying mechanism of PEI-GNP–induced liver injury in mice, we further determined the gene expression of pro-inflammatory cytokines in the liver (Figure 3). Hepatic mRNA expression of the pro-inflammatory cytokines including tumor necrosis factor alpha (*Tnf-*α), interleukin-6 (*Il-6*), and *IL*-*1*β were significantly increased in mice treated with PEI-GNPs at 23 μg/mouse for 1 week, and such inflammatory responses were not found in mice treated with PEI-GNPs at 23 μg/mouse for 24 h, and 11.5 μg/mouse for 24 h and 1 week. Meanwhile, the level of *Il-10* mRNA was comparable in all groups. These results indicated that hepatic deposition of PEI-GNPs was associated with the inflammation-mediated liver injury.

### The Effects of Polyethyleneimine–Gold Nanoparticles on the Expression of Hepatic Drug Transporters in Mice

**FIGURE 4:**
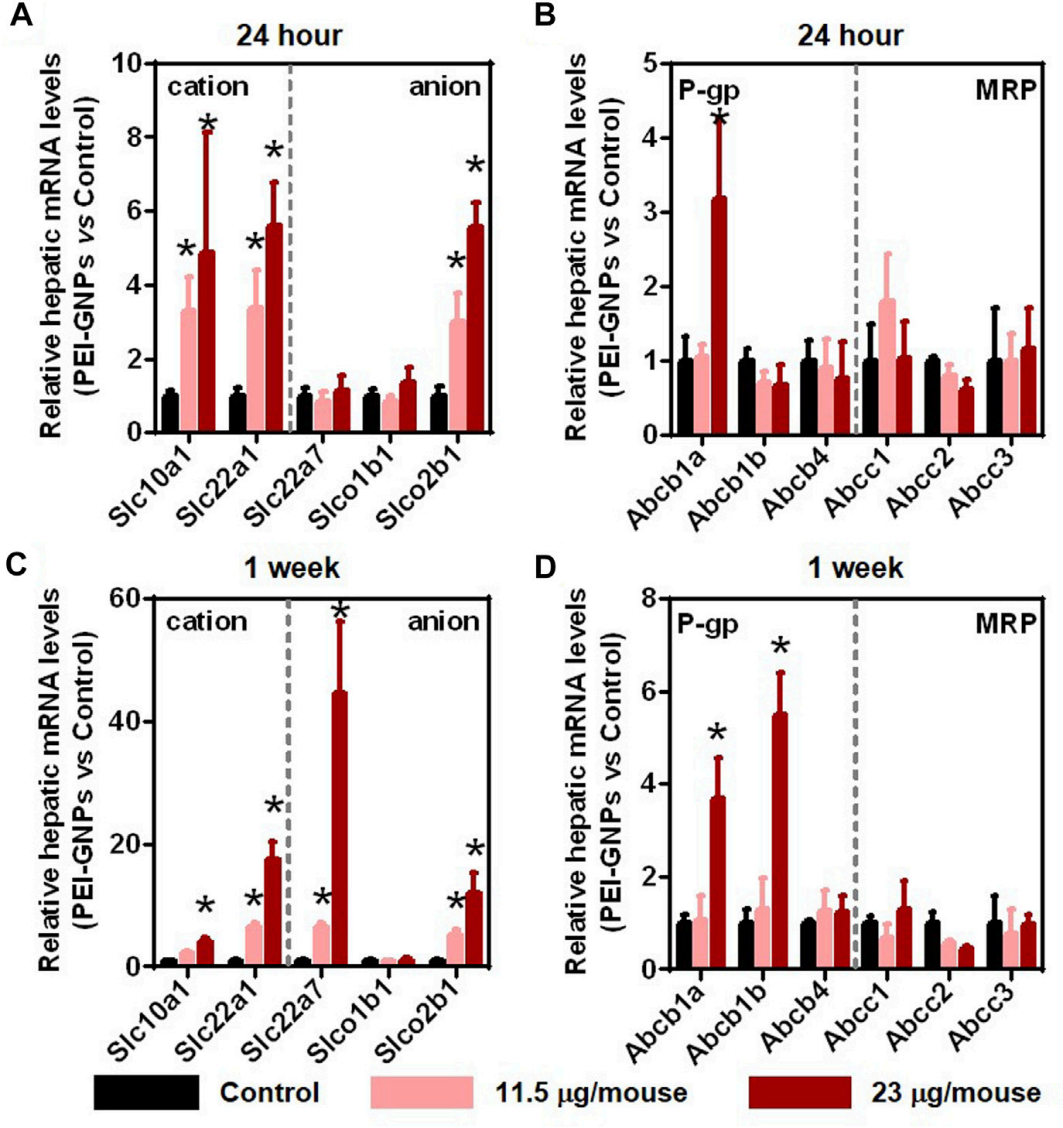
Effect of PEI-GNPs on the activity of hepatic drug transporters in mice after treatment for 24 h **(A–B)** and 1 week **(C–D)**. The typical genes involved in drug uptake **(A, C)** and efflux **(B, D)** transporters in the liver of mice treated with PEI-GNPs. Each bar represents mean ± SD from 6 mice. *p < 0.05 vs. the mice treated with PBS.

Previous studies have demonstrated that injected GNPs were mainly trapped in the liver and were not excreted from the liver even after 28 days postinjection (Li et al., 2020; Zhou S. et al., 2020). Drug transporters in the liver play an important role in the uptake and efflux of xenobiotics (Almazroo et al., 2017). In order to investigate the potential mechanism of PEI-GNP–mediated liver inflammation, we further analyzed the gene expression of drug uptake and efflux transporters (Figure 4). After treatment with PEI-GNPs, the hepatic expression of the genes *Slc22a1* and *Slc10a1*, involved in the uptake of cationic xenobiotics, *Slco2b1*, an anionic drug transporter, and *Abcb1a*, mediated the hepatic elimination through P-glycoprotein (P-gp), were increased in PEI-GNP–treated mice in a dose-dependent manner. The mRNA expression of *Slc22a7* significantly increased 1 week postinjection of PEI-GNPs and was not changed after 24 h of PEI-GNP treatment at the dose of 11.5 and 23 μg/mouse. The gene expression of *Abcb1b* was obviously increased in PEI-GNP–treated mice at the dose of 23 μg/mouse for 1 week. The expression of the genes, including *Abcc1*, *Abcc2*, and *Abcc3*, the multidrug resistance–related protein (MRP), *Slco1b1*, and *Abcb4* were comparable in all groups. These results highlighted the evidence that the increased expression of genes involved in hepatic uptake and efflux transporters was associated with PEI-GNP deposition-mediated liver inflammation and injury in mice.

### The Effect of Polyethyleneimine–Gold Nanoparticles on the Activation of Drug-Metabolic Enzymes in Mice

**FIGURE 5:**
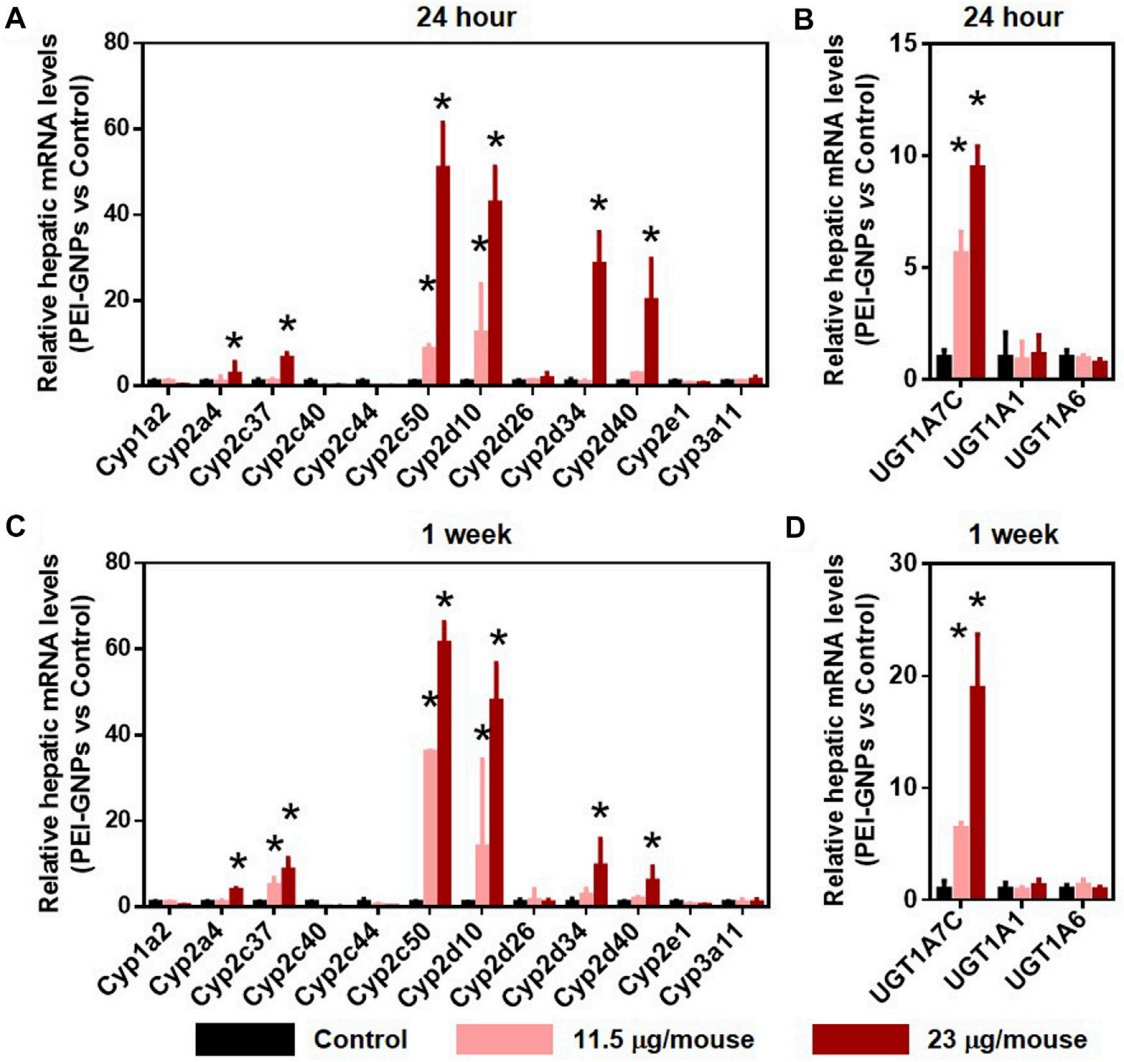
Effect of PEI-GNPs on the gene expression of drug-metabolizing enzyme in the liver of the mice after 24-h and 1-week treatment. Hepatic mRNA levels of CYP450 **(A, C)** and UGT **(B, D)** isoforms in response to PEI-GNP administration for 24 h **(A–B)** and 1 week **(C–D)**. All the data are presented as mean ± SD. *n* = 6. *p < 0.05 vs. the mice treated with PBS.

Cytochrome P450 (CYP450), the well-known Phase I drug-metabolic enzyme, is responsible for the biotransformation and metabolism of more than 75% of all marketed drugs (Almazroo et al., 2017). UDP-glucuronosyltransferases (UGTs) are involved in the elimination of the drugs or metabolites by enzymatically conjugating with hydrophilic endogenous compounds (Almazroo et al., 2017). In Figure 5, PEI-GNP treatment for 24 h and 1 week showed the strong induction of the expression of CYP450 isoforms, such as *Cyp2a4*, *Cyp2c37*, *Cyp2c50*, *Cyp2d10*, *Cyp2d34*, and *Cyp2d40*, in a dose-dependent manner. Similarly, induction of the genes involved in UGT-mediated hepatic metabolism, such as *Ugt1a7c*, was observed in PEI-GNP–treated mice. These results suggested that the alteration of the function involved in normal drug-metabolic enzymes may be a driver of nanoparticle-induced liver inflammation and hepatoxicity.

### The Effect of Polyethyleneimine–Gold Nanoparticles on the Hepatic Lipogenesis and Gluconeogenesis in Mice

**FIGURE 6:**
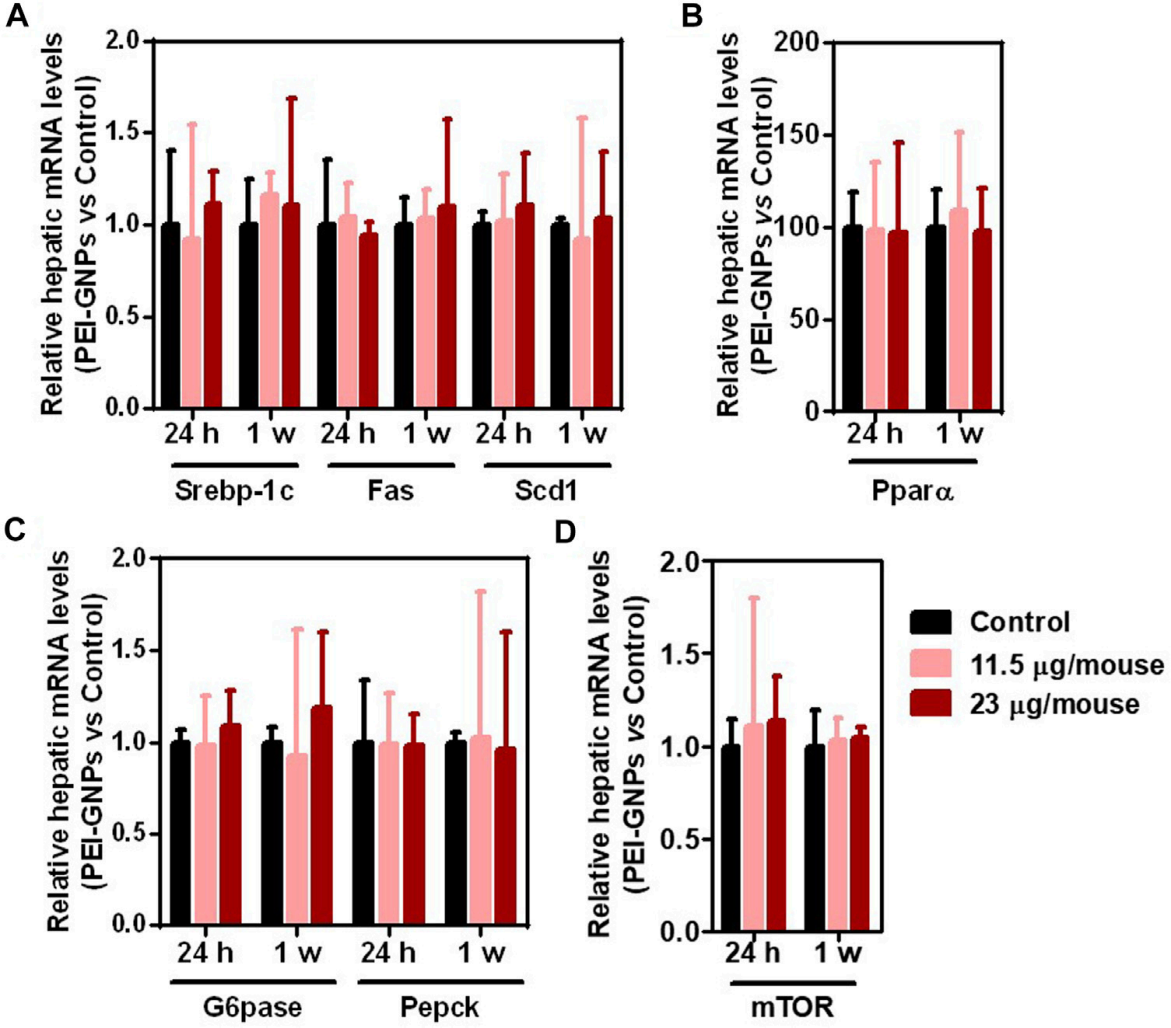
Effect of PEI-GNPs on *de novo* lipogenesis, fatty acid oxidation, and gluconeogenesis in mice. **(A)** The mRNA expression of the representative genes encoded *de novo* lipogenesis including *Srebp-1c*, and its targeting genes, such as *Fas* and *Scd1*, in the liver of PEI-GNP–treated mice for 24 h and 1 week. **(B)** The expression of key genes involving fatty acid oxidation, such as *Ppar*α, in the liver of PEI-GNP–treated mice. **(C)** Gene expression of gluconeogenesis including *G6pase* and *Pepck* measured by real-time PCR. **(D)** Hepatic mRNA level of *mTOR* in mice after treatment with PEI-GNPs for 24 h and 1 week (*n* = 6).

Recent studies have reported that disturbance to the drug metabolic enzyme is observed in patients with NAFLD (Papatheodoridi et al., 2020; Zhou S. et al., 2020). Studies have demonstrated that the continuous fructose consumption leads to increased *de novo* lipogenesis and gluconeogenesis, and even NAFLD in both humans and rodents (Stanhope 2012; Karise et al., 2017; Zhou F. et al., 2020). The mRNA expressions of *de novo* lipogenesis including *Srebp-1c* and its target genes, including fatty acid synthase (*Fas*) and stearoyl coenzyme A desaturase 1 (*Scd1*), fatty acid oxidation including peroxisome proliferator–activated receptor alpha (*Ppar*α), gluconeogenesis including glucose-6-phosphatase (*G6pase*) and phosphoenolpyruvatecarboxykinase (*Pepck*), and nutrient sensor including mechanistic target of rapamycin (mTOR) were comparable in all groups (Figure 6).

### Cell Viability of Polyethyleneimine–Gold Nanoparticles in HepaRG Cells

**FIGURE 7:**
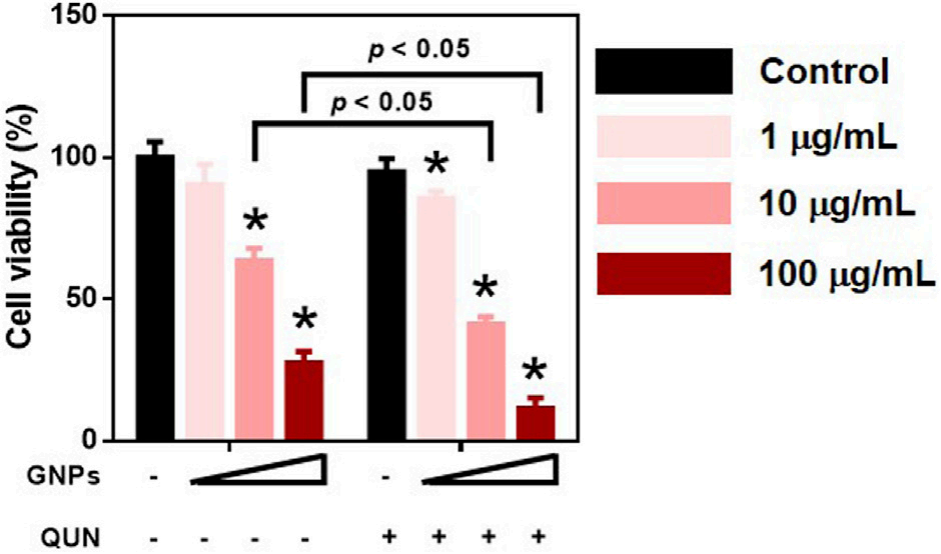
Effect of 10 μM quinidine (QUN) pretreatment on cell viability in HepaRG cells after treatment with PEI-GNPs at the doses of 1, 10, and 100 μg/ml for 24 h. *p < 0.05 vs. the cells treated with PBS.

The cytotoxicity of PEI-GNPs for HepaRG cells was determined using the CCK-8 assay. PEI-GNPs showed significant decrease of cell viability at the doses of 10 and 100 μg/ml for 24 h (Figure 7). Quinidine (QUN) has been reported to decrease the hepatic drug clearance by inhibiting the drug-metabolizing enzyme CYP450 (Gessner et al., 2019). QUN pretreatment significantly decreased the viability of HepaRG cells treated with GNPs at the doses of 1, 10, and 100 μg/ml. These data suggest that the GNP–liver interaction plays the vital role in PEI-GNP–induced hepatotoxicity.

## Discussions

Due to the unique optical and thermal characteristics along with their tunable size and surface chemistry, gold nanoparticles (GNPs) have been used as a powerful delivery platform for drugs, peptides, proteins, and RNA molecules (Fan et al., 2020; Zhang et al., 2020). Recently, GNPs have been reported to realize photoacoustic imaging–guided complementary photothermal or gene therapy for cancer through modification of polycationic chitosan (Dai et al., 2021). However, despite great interest in their biomedical application, there are only few clinical trials or drugs of GNPs approved by the U.S. Food and Drug Administration (FDA) (Bobo et al., 2016; Wong et al., 2020). It has been reported that smaller sized GNPs induced more inflammatory responses, cytotoxic reactions, DNA double-strand disruptions, oxidative stress, apoptosis, and venous intimal disturbance than larger sized GNPs (Abdelhalim et al., 2018). Our recent study has demonstrated that GNPs could interact with hepatocytes, liver sinusoidal endothelial cells, and Kupffer cells after intravenous injection, which resulted in the hepatic deposition of GNPs (Li et al., 2020). Intraperitoneal injection of 10 nm GNPs for 1 week at the dose of 12.5 mg/d dramatically damaged the liver function, elevated the hepatic lipid biomarker MDA, and promoted the generation of oxidative stress in rats, indicating the potential hepatotoxicity induced by GNPs (Abdelhalim et al., 2018). So it is important to address a better understanding of the possible mechanism of hepatic metabolism and transport of the deposited GNPs.

ICR mice have been used in many scientific research fields including pharmacology, toxicity, and pharmaceutical product safety testing for decades (Kim et al., 2017). In this study, we explored the effect of GNPs modified with polyethyleneimine (PEI) on liver inflammation, function of hepatic drug-metabolic enzymes, lipid metabolism, and gluconeogenesis in male ICR mice after intravenous injection for 24 h and 1 week at the doses of 11.5 and 23 μg/mouse. PEI has been introduced as a reagent for nucleic acid delivery by protecting RNA from enzymatic and nonenzymatic degradation during transferring across the cell membrane (Jia et al., 2019). Recently, PEIs have attracted great interest in the modification of nanoparticles to increase the loading capacity due to their special characteristics of structure, branched internal cavity, and abundant terminal amines (Chen et al., 2020). PEI at the low molecular weight (0.6, 1.2, and 1.8 kDa) showed better degradable properties, lower toxicity, and transfection efficiency than PEI at the high molecular weight (10 and 25 kDa). Nevertheless, when PEI is at the high molecular weight, its nondegradable properties and high cytotoxicity have hampered its biomedical application. Herein, GNPs grafted with 10 kDa PEI induced significant liver injury in mice at the dose of 23 μg/mouse for 1 week, including significant alterations in biochemical parameters, obvious increase in the gene expression of pro-inflammatory cytokines, and disruption in the expression of hepatic drug-metabolic enzymes. Moreover, the deposited PEI-GNPs did not induce significant hepatic steatosis and gluconeogenesis in mice.

Hepatic inflammation is considered as the vital driver of drug-induced liver injury and nanoparticle-mediated hepatotoxicity (Chen et al., 2015; Zhu et al., 2021). Inflammatory responses in the liver will increase the risk of the development and progression of liver diseases, including nonalcoholic fatty liver disease (NAFLD) and alcoholic liver disease (ALD) (Chen et al., 2018; Kazankov et al., 2019). The long retention time of the deposited GNPs in the liver induced significant liver injury, which is associated with GNP-induced inflammation or immunological responses (Li et al., 2020). The results obtained from real-time qPCR showed that mice treated with PEI-GNPs exhibited obvious liver inflammation, such as the increased expression of inflammation-associated genes including *Tnf-*α, *Il-6*, and *IL*-*1*β. Therefore, GNPs modified with PEI-induced hepatoxicity were associated with the increased expression of hepatic inflammatory cytokines.

The liver is a major organ in the metabolization, biotransformation, and detoxification of drugs and exogenous substances (Almazroo et al., 2017). In the clinical medical practice, disturbance in the function of hepatic drug-metabolic enzymes could induce hepatic inflammation and the damage of hepatocyte function, which is the major cause of drug-induced liver injury or acute liver failure (Lee 2013; Zhang et al., 2019). Hepatic uptake transporters, such as solute carrier (SLC), and efflux transporters, including ATP-binding cassette (ABC), contribute to regulate the absorption, distribution, metabolism, and excretion of endogenous or xenobiotics *in vivo* (Almazroo et al., 2017; Zhang et al., 2011). Cytochrome P450 (CYP450) enzymes, mainly expressed in the liver, are involved in the hepatic biotransformation and metabolism of xenobiotic substances, and disruption in the function of CYP450 changed the pharmacokinetics and pharmacodynamics of drugs and increased the risk of drug-induced liver injury (Almazroo et al., 2017; Malki and Pearson 2020). UDP-glucuronosyltransferase (UGT) is the well-known Phase II drug-metabolic enzyme involved in the elimination of drugs or their metabolites (Almazroo et al., 2017). The hepatic gene expression of drug-metabolic enzymes, including *Slc22a1*, *Slc10a1*, *Slco2b1*, *Abcb1a*, *Slc22a7*, *Cyp2a4*, *Cyp2c37*, *Cyp2c50*, *Cyp2d10*, *Cyp2d34*, *Cyp2d40*, and *Ugt1a7c*, was increased in PEI-GNP–treated mice, and no significant changes in the genes, such as *Abcc1*, *Abcc2*, *Abcc3*, *Slco1b1*, *Abcb4*, *Cyp1a2*, *Cyp2c40*, *Cyp2c44*, *Cyp2d26*, *Cyp2e1*, *Cyp3a11*, *Ugt1a1*, and *Ugt1a6*, were observed in PEI-GNP–treated mice. Collectively, the evidence obtained from real-time PCR analysis in this study indicated that the deposited PEI-GNPs in the liver induced hepatotoxicity due to the disturbance in the function of drug-metabolic enzymes, which may be an early hepatic detoxification of nanomaterial–liver interaction.

## Conclusion

Herein, we explore the potential hepatic impact of GNPs modified with PEI in mice after intravenous injection at the doses of 11.5 and 23 μg/mouse for 24 h and 1 week, respectively. The results provide the evidence that PEI-GNPs deposited in the liver do not change the liver function, and induce hepatic lipid accumulation and gluconeogenesis. However, PEI-GNP accumulation in the liver is associated with increased liver inflammation, as evidenced by the gene expression of pro-inflammatory cytokines. Moreover, the GNP-induced hepatotoxicity in mice is in partly due to liver inflammation–triggered disruption in the function of drug-metabolic enzymes, including hepatic uptake and efflux transporters, CYP450 and UGTs, respectively. The study provides evidence that it is necessary to consider the nanomaterial–liver interaction and manipulate the surface chemistry of GNPs prior to biomedical application of nanoparticles.

## Data Availability

The original contributions presented in the study are included in the article/Supplementary Material; further inquiries can be directed to the corresponding authors.
